# Kombucha production using raw materials from Brazil

**DOI:** 10.3389/fnut.2026.1770255

**Published:** 2026-06-17

**Authors:** Eduardo Leonarski, Guilherme Dallarmi Sorita, Karina Cesca, Débora de Oliveira

**Affiliations:** Department of Chemical Engineering and Food Engineering, Federal University of Santa Catarina (UFSC), Florianópolis, Santa Catarina, Brazil

**Keywords:** agro-industrial by-products, bioactive compounds, fruits, herbs, phenolic compounds

## Abstract

Kombucha production has increased significantly in recent years, and analog beverages (fermented with extracts other than *C. sinensis* tea) are also gaining market share. Brazil, one of the largest fruit-producing countries with vast fruit diversity, has expanded their research fields to develop new products, including kombucha. This literature review aims to present studies being conducted in Brazil on the production of traditional kombucha (with green or black tea) and analog extracts. Based on the results, it was observed that a large part of the analog beverage production is carried out with fruits mainly from the Northeast and South regions of Brazil. However, it is also done with other types of extracts, such as coffee, yerba mate, and yams. In addition, some studies have used byproducts from cocoa (*Theobroma cacao*), acerola, guava, tamarind, as well as mango and grape peel. It was also observed that during fermentation, regardless of the type of extract, both total phenolic compounds and antioxidant activity tend to increase. Although regulations for kombucha production in Brazil have already been established, some challenges remain regarding the use of tea and SCOBY, demystifying probiotic effects (since this is not yet regulated), uncontrolled ethanol production, and the need for specific legislation for secondary fermentation. Overall, Brazil shows great potential for developing new products, such as kombucha-type beverages, where the fermentation process is similar to that of traditional kombucha; however, regulating the process using alternative extracts and SCOBY (which may present a consortium of different microorganisms according to regions) remains a challenge for achieving homogeneous, feasible results.

## 1 Introduction

Kombucha is currently popular worldwide, being a fermented beverage that originated in Asia many years ago, about 220 B.C (1, 2). The symbiotic culture of bacteria and yeast (SCOBY) is responsible for the fermentation process, using green or black tea (*Camellia sinensis*) as an extract for kombucha fermentation, which results in a refreshing, bittersweet, and lightly carbonated drink. The characteristics of kombucha can vary according to several factors, such as the type of tea or raw material, the microorganisms present in the SCOBY, and the fermentation time and temperature (3, 4).

Although much of kombucha production is done at home by consumers, due to the ease and simplicity of the fermentation process, numerous industries have adapted and scaled up the fermentation and production process of the beverage (4). As the fermentation process can vary according to the added SCOBY, some legislation has been published in recent years to regulate the final characteristics of the beverage (5–7).

The regulations published by the Ministry of Agriculture, Livestock and Supply (MAPA) (6), the sector responsible for the inspection of kombucha beverages in Brazil, established values for pH (between 2.5 and 4.2), volatile acidity (between 30 and 130 mEq), alcohol content (maximum 0.5% v/v for non-alcoholic beverages and between 0.6% and 8.0% v/v for alcoholic beverages), and pressure in kombucha with added CO_2_ (between 1.1 and 3.9 atm at 20 °C). According to the legislation, in addition to the mandatory ingredients (potable water, *Camellia sinensis* tea, sugar and the SCOBY), it is possible to add spices and infusions of plant species in water or their extracts, authorized by specific legislation, fruits, vegetables, honey, molasses and other sugars of vegetable origin, fibers, vitamins, mineral salts, natural flavoring additives and natural colorings and other nutrients approved by specific legislation, industrially pure CO_2_ and new ingredients approved by the Brazilian National Health Surveillance Agency (ANVISA).

According to these specifications and the fact that Brazil is one of the countries with the highest production of fruits, only behind India and China (8), many studies have been carried out in recent years aiming at the use of alternative raw materials to produce kombucha-type (or analogs) beverages. Based on these observations, the objective of this review is to address studies on the production of traditional kombucha (with *Camellia sinensis* tea) and its analogs (adding other extracts as raw material) from Brazil, evaluating the production process, fermentation kinetics (sugar consumption and production of organic compounds, as well as the evaluation of bioactive compounds), and sensory analysis of the beverages.

## 2 Kombucha production

The kombucha market has experienced significant growth in production volume and product variety, gaining popularity in Western cultures as an alternative to traditional soft drinks due to its lower calorie count and superior nutritional composition (9, 10). The beverage production process is simple; basically, green or black tea is infused, followed by filtration. Afterward, sugar is added, and it is allowed to cool (~25 °C) before finally adding the inoculum (v/v) or the SCOBY (w/v), or both (this may vary depending on the manufacturer) (3, 4, 11). To produce analogous beverages, the preparation of the alternative raw material may require different unit operations, as described by Leonarski et al. (3), including sanitization, drying, disintegration, pulping, and sterilization, among others. These analogous beverages (produced with alternative extracts to *Camellia sinensis* tea) are generally called kombucha-type beverages.

In Brazil in 2020, approximately 50 kombucha companies were members of the Brazilian Kombucha Association (ABKOM), which was created in 2018, generating approximately $ 2 million in revenue (10). According to the Ministry of Agriculture and Livestock (MAPA) (12), from 2019 (when kombucha production regulation was introduced) to 2025, production increased by 923%, with 249 manufacturers; the state of São Paulo stood out with 42. Furthermore, according to Saito et al. (13), eleven patents were identified in Brazil, most of which were related to the beverage's production process and its use in the production of other products (e.g., ice cream).

Table 1 presents studies conducted in Brazil on the production of traditional kombucha (using green or black tea as raw material). It can be observed that, in these studies, green tea is generally preferred (83.3%) over black tea (41.7%), and concentrations range from 0.5 to 3.0% (w/v). The most used method for obtaining the tea is boiling water (41.7% of studies), followed by 95 °C (25% of studies). However, several other temperatures were used (70 °C, 75 °C, 85 °C, 90 °C, and 121 °C, each with only one study, corresponding to 8.3% each). Regarding sugar content, concentrations ranging from 30 to 100 g/L are used, with 50 g/L the most common (33.3%), followed by 80 g/L (16.7%) and 100 g/L (16.7%).

**Table 1:** Materials and parameters used for the fermentation of traditional kombucha from *Camellia sinensis* (green tea or black tea).

Extract	Infusion	Initial sugar (g/L)	Inoculum (v/v) and SCOBY content (w/v)	Process parameters	References
Green or black tea (1.5% w/v)	Boiling mineral water for 15 min	80	10% (v/v) and 5% (w/v)	28 °C for 15 days	(14)
Green or black tea (1.2% w/v)	Infusion with water at 75 °C for 2 min (green tea) and at 95 °C for 4 min (black tea)	50	10% (v/v) and 3% (w/v)	25 °C for 10 days	(15)
Green tea (0.5% w/v)	Boiling water	50	1% (v/v) and 2.5% (w/v)	24 °C for 14 days	(16)
Green tea (12.5% w/v)	Boling water for 15 min	75	20% (v/v) and 3% (w/v)	25 °C for 7 days	(17)
Green tea (0.5% w/v)	121 °C for 15 min	70	10% (v/v) and 4% (w/v)	30 °C for 15 days	(18)
Black tea (1.2% w/v)	Infusion with water at 95 °C for 4 min	50	10% (v/v) and 3% (w/v)	25 °C for 10 days	(19)
Black tea (3% w/v)	95 °C for 10 min	100	10% (v/v) and 2.5% (w/v)	23 °C for 9 days	(20)
Green tea (0.5% w/v)	100 °C for 10 min	100	30% (w/v)	25 °C for 10 days	(21)
Green tea (1.2% w/v)	70 °C for 1 min	50	10% (v/v) and 3% (w/v)	25 °C for 5 days	(22)
Green tea (1.0% w/v)	90 °C for 15 min	30	10% (v/v)	28 °C for 15 days	(23)
Green tea (1.2% w/v)	85 °C for 15 min	65	10% (v/v)	28 °C for 12 days	(24)
Green, white and black tea (0.8% w/v)	Boiling water for 10 min	80	30% (v/v)	28–30 °C for 10 days	(25)

For inoculants, this can be added as a “starter tea”, that is, previously fermented kombucha (in liquid form, v/v), or as a SCOBY (cellulosic form containing the bacteria and yeasts) in solid form (w/v). The concentration ranges from 1 to 30% (v/v) in liquid form, with 10% (v/v) predominant, corresponding to 66.7% of studies, and for SCOBY, the concentration ranges from 2.5 to 5.0% (w/v), with concentrations of 2.5% (25%) and 3% (50%) being most used. Fermentation lasted from 5 to 15 days, predominating on days 10 (33.3%) and 15 (25%), with temperatures ranging from 23 to 30 °C, with temperatures of 25 °C (41.7%) being predominant, followed by 28 °C (33.3%) and 30 °C (16.7%).

The ingredient concentrations and fermentation parameters presented in Table 1 are similar to those reported in other reviews on kombucha production (2, 3, 9). Furthermore, according to the Department of Agriculture and Markets of New York (USA) (5), the addition of 4–5 g/L of tea is suggested, with most studies adding higher concentrations. Regarding the amount of sugar (or other carbohydrate sources) used, an addition between 5%−15% (w/v) is suggested, with only one study adding a lower concentration (3%, w/v) (7) Furthermore, fermentation temperatures between 22-32 °C are recommended by Kombucha Brewers International (26), with a suggested fermentation time of 7–10 days; however, this duration mainly depends on the final characteristics of the beverage obtained during fermentation (4–26).

## 3 Kombucha from unconventional substrates

A search of the SCOPUS database conducted in December 2025, using the keywords “kombucha” AND “fruits” yielded 185 documents, including 122 research articles and 30 reviews. These articles were then analyzed by reviewing the titles and abstracts of studies conducted in Brazil, resulting in the selection of 27 articles (7 reviews). Of these 20 articles, 15 were selected for the review study. During the research, 3 additional articles on yerba mate were added, along with other articles found while writing the review article, bringing the total to 20 research articles that utilize unconventional substrates for kombucha production in Brazil.

The research was also conducted in other databases, such as Web of Science and PubMed. However, both databases yielded fewer articles: Web of Science found 51 documents (43 research papers and 8 reviews), while PubMed found only 18 (7 of which were reviews). All the articles found were already included in the search conducted using the SCOPUS database.

**Figure 1:**
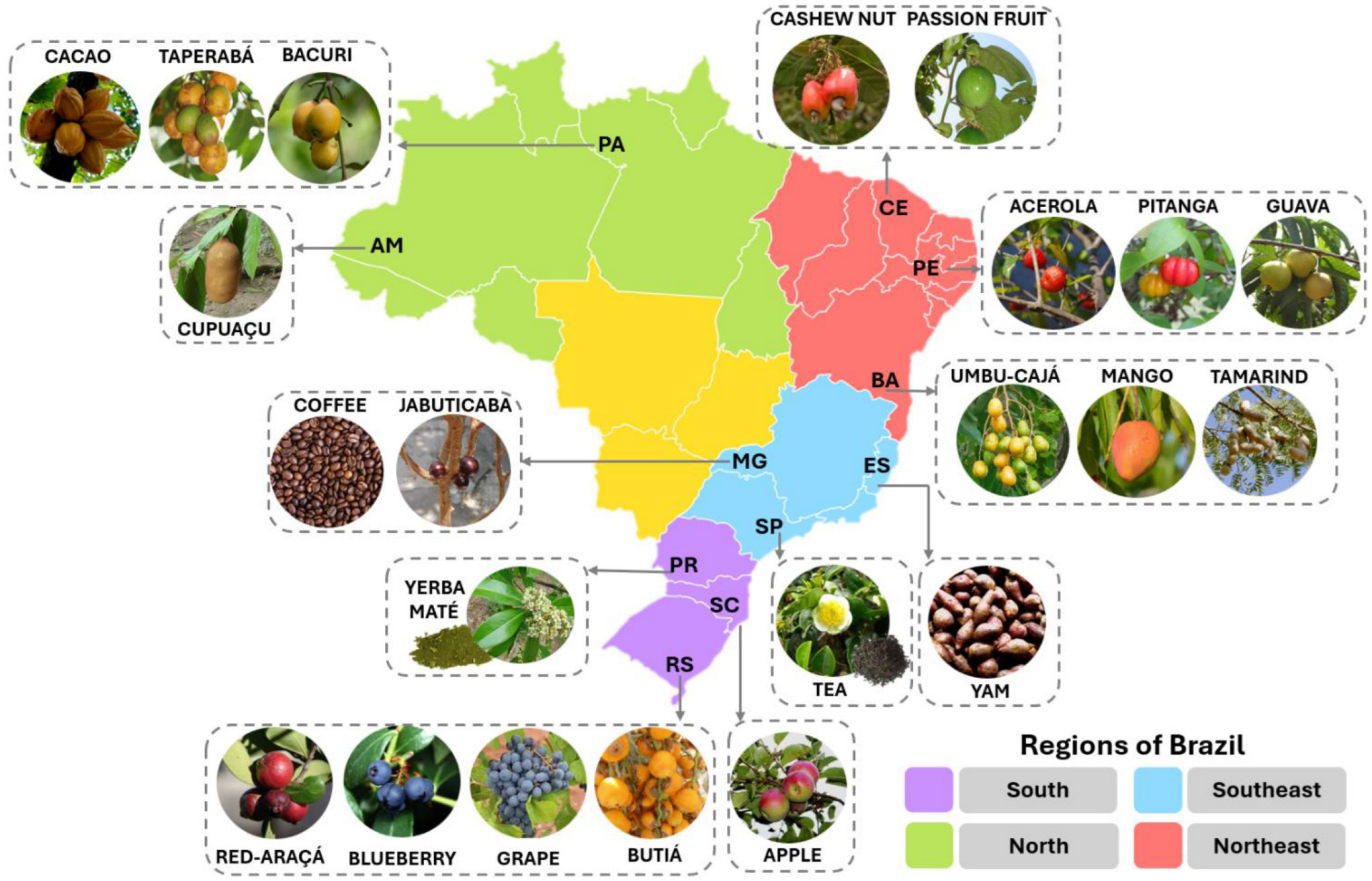
Raw materials to produce kombucha and kombucha-like beverages from Brazilian regions.

As mentioned earlier, Brazil is renowned for its diverse array of fruit species. Figure 1 shows the regions of Brazil where the raw materials used in the production of kombucha and similar beverages are predominantly produced. As can be observed, except for the central-west region, all the others have raw materials that were used. The production of *Camellia sinensis* tea primarily occurs in the state of São Paulo (SP), while the Southeast also stands out to produce yams in the state of Espírito Santo (ES), coffee, and jabuticaba in the state of Minas Gerais (MG). In the South region, the state of Paraná (PR) stands out to produce yerba mate, Santa Catarina (SC) for apple production, while Rio Grande do Sul (RS) stands out to produce red guava, blueberry, grape, and butiá, being the state with the largest quantity of raw materials used for beverage production (27).

The Northeast region stood out with the greatest variety of raw materials used, with umbu-cajá, mango, and tamarind coming from the state of Bahia (BA), acerola, pitanga, and guava from the state of Pernambuco (PE), and cashew nut and passion fruit from the state of Ceará (CE). In the North region, the state of Pará (PA) stood out, using cocoa, taperabá, and bacuri, while cupuaçu was used in the state of Amazonas (AM) (27). It is essential to emphasize that the utilization of native Brazilian fruits enhances the region's value and, consequently, generates income. Therefore, it is crucial to conduct studies to explore new sources for producing kombucha-type beverages, thereby promoting regional development throughout the country.

As shown in Table 2, most kombucha-like beverages utilize different types of fruit as raw materials, with two studies using pitanga (17, 28) and two using passion fruit (21, 24). In addition to these fruits, 15 other types of fruits or their byproducts were used. Byproducts used included those from cocoa (*Theobroma cacao*) (29), acerola (30, 31), grava and tamarind (31), as well as mango and grape peel (23). Furthermore, yerba mate was used in four different studies (28, 32–34), while coffee was used in two studies (20, 35) and one study that used coffee leaves (36).

**Table 2:** Materials and parameters used for kombucha like-beverages (or analogs) fermentation with alternative raw material.

Raw preparation	Extract	Initial sugar (g/L)	Inoculum (v/v) and SCOBY content (w/v)	Process parameters	References
Sanitized pitanga and umbu-cajá were pulped manually	Pitanga and umbu-cajá (15% v/v)	–	Second fermentation	25 °C for 48 h	(17)
Acerola byproduct pasteurized (121 °C/15 min)	Acerola byproduct (1%, 3%, and 5% w/v)	70	10% (v/v) and 3% (w/v)	30 °C for 15 days	(30)
Yerba-maté infusion (80 °C for 10 min)	Yerba-mate tea (0.5%, 0.75%, and 1.0%, w/v)	50	10% (v/v)	20 °C, 25 °C, and 30 °C for 12 days	(32)
The flowers were washed and dried (60 °C)	Wax mallow flower extract (0.5%, w/v)	50	1% (v/v) and 2.5% (w/v)	24 °C for 14 days	(16)
Bleaching (100 °C/3 min) and homogenized	Blueberry (10%, w/v)	–	6% (w/v)	Room temperature for 21 days	(37)
Pasteurized cashew nut extract (63 °C/30 min)	Cashew nut beverage (100% v/v)	–	Second fermentation 10% (v/v)	28 °C for 72 h	(38)
Coffee infusion (5 min)	Coffee (2%, w/v)	50	10% (v/v) and 5% (w/v)	25 °C for 21 days	(35)
Coffee infusion (95 °C/10 min)	Coffee (3%, w/v)	100	10% (v/v) and 2.5% (w/v)	23 °C for 9 days	(20)
Pre-cooked at 60 °C/10 min, then crushed and homogenized	Yam extract (10% and 20%, w/v)	50	10% (v/v)	28 °C for 5 days	(39)
Infusion in water at90 °C/5 min	Acerola, guava or tamarind by-products (10% w/v)	–	15% (v/v) and 20% (w/v)	Room temperature for 14 days	(31)
100 g of the nectars and then adding water and sugar	Bacuri (B), Taperebá (T), and Cupuaçu (C) nectars (40%−46% w/v)	B: 100 T: 30 C: 18.2	Second fermentation	Room temperature for 24–36 h	(40)
Green tea (GT) and Yerbá-mate (YM) infusion at 80 °C/10 min	T1: 1.0% GT; T2: 0.75% GT and 0.25% YM, and T3: 0.5% GT and 0.5% YM	50	10% (v/v)	20 °C for 9 days	(33)
Coffea arabica leaf tea (CL) and toasted maté (TM) infusions at 95 °C/10 min	CL and CL-TM (50:50) (2.5%,w/v)	100	10% (v/v) and 2.5% (w/v)	23 °C for 9 days	(36)
Roasted yerba-maté leaves infusion at 100 °C/10 min	Roasted yerba-maté tea (1.2%, w/v)	~23	20% (v/v) and 8% (w/v)	25 °C for 20 days	(34)
Passion (P) and Apple (A) fruits were washed and sanitized	Passion fruit pulp (20% w/v), Apple fruit (100% w,v)	100	30% (w/v)	Room temperature for 10 days	(21)
Passion fruit leaf infusion (85 °C/15 min)	*Passiflora edulis* tea (1.2% w/v)	65	10% (v/v)	28 °C for 12 days	(24)
Suspending the fruit residue powder (1:8, powder:water)	Grape (GKP) and mango (MKP) peel extracts (20%, v/v)	–	Second fermentation	28 °C for 48 h	(23)
Cocoa beans (CB) were roasted (180 °C/15 min), then cracked and peeled. CB was wet sterilized at 121 °C/15 min	CBS in water at 85 °C ofr 10 min (0.7 %, 1.5 %, and 2.3 %, w/v)	80	6.5% (v/v) and 10% (w/v)	25 °C for 24 h	(29)
Pitanga nectar was prepared with 60% (w/v) water. Yerba mate infusions at 80 °C/7.5 min	Yerba-maté tea (0.4%, 0.8%, and 1.2% w/v); and pitanga pulp with yerba mate infusion (30:70, v/v)	50–150	10% (v/v)	20–30 °C for 15 days	(28)
The pulps (red-araçá, butiá-da-serra, and jaboticaba) were blended in a blender until fully disintegrated	Red-araçá, Butiá-da-serra, and Jaboticaba pulps (10% w/v)	70	10% (v/v)	25 °C for 10 days	(43)

Sample preparation depends on the type of raw material used, as already reported in the review by Leonarski et al. (3). In the case of other types of teas or leaves, the material is usually dried, crushed, and an infusion is made to obtain the tea. In the case of coffee, the infusion is made directly. In the case of fruits, the pulp is usually removed, and depending on the fruit, it may also be disintegrated, and finally, nectar is obtained (water and/or sugar may be added). For fruit by-products, a sterilization step is recommended to ensure consumer safety.

Regarding kombucha production using alternative extracts, extract concentrations vary widely depending on the fruit used (Table 2). The concentration of added sugar also varies considerably, with 50 g/L (30% of studies) or 100 g/L (25% of studies) being the most common. In some studies (25%), no sugar is added, as it is derived from the raw material itself (17, 23, 31, 37, 38). Regarding the inoculum (v/v), concentrations range from 1 to 20%, with 10% being the predominant addition (73.3% of studies). The addition of SCOBY varied between 2.5 and 30%, with 2.5% being the most common addition (30%) in the studies.

Concerning the time and temperature for the fermentation of the beverages, the time ranged from 1 to 21 days, while the temperature ranged from 20 °C to 30 °C, with temperatures of 25 °C (30% of the studies), 28 °C (25% of the studies), and room temperature (20%) being predominant. It is interesting to note that when making kombucha analogs, some studies have performed a second fermentation (17, 23, 38, 40), that is, after fermentation with traditional kombucha, a new fermentation is carried out, now adding the alternative extract as raw material, which generally has a short duration, from 24 to 72 h.

## 4 Characteristics of microorganisms in tea fungus

Kombucha fermentation occurs through a Symbiotic Culture of Bacteria and Yeast (SCOBY), in which the yeast is initially responsible for the hydrolysis of sucrose and then converts the resulting metabolites (glucose and fructose) into ethanol and CO_2_. The bacteria are responsible for oxidizing glucose into various acids, including acetic, gluconic, and glucuronic acids, as well as producing bacterial cellulose, aided by several enzymes essential for biosynthesis (3, 41). The main yeasts present in SCOBY are usually from the genera *Brettanomyces, Zygosaccharomyces, Saccharomyces*, and *Pichia*. At the same time, the bacteria are acetic acid bacteria (AAB), typically from the genus *Komagataeibacter* spp., but may also contain lactic acid bacteria (LAB) (3, 4, 42).

Among the studies presented in Tables 1, 2, only seven studies identified the microorganisms present in SCOBY. Of these studies, six identified the yeast *Brettanomyces bruxellensis* (18, 20, 29, 30, 36, 39), while the study of Suhre (43) identified the yeasts *Brettanomyces anomalus* and *Zygosaccharomyces* sp. Two studies identified *Zygosaccharomyces bisporus* (18, 30) and yeasts from the *Pichiaceae* spp. family were commonly reported (18, 20, 29, 30, 36, 39). Regarding bacteria, the majority reported were from the family *Acetobacteraceae*, in particular *Komagataeibacter* sp., mainly *K. rhaeticus, K. saccharivorans, K. sucrofermentans, K. europaeus, K. intermedius*, and *K. xylinus*, among others. Two lactic acid bacteria, *Latilactobacillus sakei* and *Pediococcus pentos*, were also identified (20, 36).

Two studies evaluated kombucha fermentation using the minimum number of microorganisms possible to standardize the process. According to the study by Laeli et al. (44), three strains are sufficient to simulate a more complex kombucha consortium, while in the study by Tran et al. (45), the authors reported that at least one yeast (such as *B. bruxellensis* and *A. indonesiensis)*, along with the tested acetic acid bacteria, is sufficient to produce the beverage closest to kombucha.

### 4.1 Kombucha fermentation kinetics

Regarding the fermentation process, several factors influence the final properties of the beverages, such as the type of extract, the consortium of microorganisms, and process parameters (temperature and time), among others. Regarding the final pH of the beverages, it is recommended, according to Brazilian legislation (6), that it be between 2.5 and 4.2, values close to those reported for traditional kombuchas made from green and black tea (between 2.5 and 3.2 after fermentation) (15, 18). The main acids formed during fermentation were acetic, glucuronic, citric, and lactic acids. The acidity and main organic acids were shown in Tables 3, 4. Regarding beverages fermented with yerba mate, when fermented with honey (34), pitanga (28), and green tea (33), they did not exceed the limit established by legislation, with values between 2.5 and 3.5. However, when only yerba mate was used, as in the study by Lopes et al. (32), the final pH value was below the established legislative limit, with values between 1.8 and 2.3 at the end of 12 days of fermentation. Acetic acid was also reported as the primary acid produced by these fermentations (28, 32, 34). Sugar consumption was only verified in the study by Santos (34), in which sugar and different honeys were used as carbohydrate sources, with sugar consumption ranging from 51.2 to 79.6% at the end of 20 days of fermentation.

**Table 3:** Acidity, organic acids, ethanol, and phenolic compounds in traditional kombucha from *Camellia sinensis* (green tea or black tea).

Extract	Acidity	Organic acids	Ethanol	Phenolic compounds	References
Green tea (GT) and black tea (BT)	GT 27.44 g/L BT 24.17 g/L	–	< 1 g/L	Gallic acid 71.04 mg/L; Caffeine 177.37 mg/L; Rutin 30.19 mg/L; Quercetin 1.22 mg/L; Catechin 8.00 mg/L	(14)
GT and BT	GT: 0.36%, BT: 0.32% (acetic acid, w/v)	Acetic: ~3 g/L; Glucoronic: GT 0.015 g/L, BT 0.02 g/L	GT: 7.29 g/L BT: 4.90 g/L	Total phenolic content (TPC): GT 0.70 mg GAE/mL, BT 1.09 mg GAE/mL; Theaflavin: GT 0.028%, BT 0.150% (w/v); Thearubigin: GT: 1.33% and BT: 1.99% (w/v)	(15)
GT	3.9 g/L	–	–	TPC: 0.56 mg/ml	(16)
GT	0.35% (acetic acid, w/v)	Acetic 0.93 g/L; Butyric 1.02 g/L; Citric 0.47 g/L; Malic 1.70 g/L; Succinic 0.39 g/L	–	Major compound: epigallocatechin gallate (EGCG) (relative percentage of the total TPC)	(17)
GT	–	Acetic 1.0 g/L; Citric 1.5 g/L; Ascorbic ~0.21 g/L	2.4 g/L	TPC: ~670 mg/L	(18)
GT	0.19% (acetic acid, w/v)	Ascorbic 0.65 mg/100 g	< 0.5%	TPC: 30.7 mg GAE/g; Anthocyanin (ANC): 0.06 mg/100 ml; Flavonoid (FL): 1.21 mg/100 ml	(21)
GT	0.20% (acetic acid, w/v)	–	4.7 g/L	TPC: 0.32 mg GAE/ml	(22)
GT	–	Acetic 2.06 g/L	0.76 g/L	TPC: ~240 mg GAE/L; FL: ~38 mg QE/L	(24)
GT	10.12 g/L		–	TPC: 306 mg GAE/g; FL: 121 mg EQ/g	(25)

**Table 4:** Acidity, organic acids, ethanol, and phenolic compounds in kombucha-like beverages using alternative raw materials.

Extract	Acidity	Organic acids	Ethanol	Phenolic compounds	References
Pitanga (PI) and Umbu-cajá (UC)	PI: 0.59%, UC: 0.70% (acetic acid m/v)	Acetic (PI-UC) 1.20–1.29 g/L; Butyric (PI-UC) 1.89–2.71 g/L; Citric (PI-UC) 2.20–0.17 g/L; Malic (UC) 0.07 g/L; Succinic (PI-UC) 0.85–0.46 g/L	–	Epigallocatechin gallate: 63.7%−76.8% of the total identified (relative quantification)	(17)
Acerola byproduct 1% (AC1), 3% (AC3), and 5% (AC5)	–	Acetic (g/L): AC1 6.58, AC3 11.98, AC5 16.38; Ascobric (g/L): AC1 0.10, AC3 0.66, AC5 0.94	AC1 9.4 g/L; AC3 8.8 g/L; AC5 10.2 g/L	TPC (mg GAE/L): AC1 265; AC3 816; AC5 983	(30)
Yerba-maté	4.8%−6.0% (acetic acid m/v)	–	–	TPC: 444–845 mg GAE/L	(32)
Flowers wax mallow	8.8 g/L	–	–	TPC: 125 mg GAE/L	(16)
Blueberry	–	–	–	TPC: 117 mg GAE/100 ml; Tannins: 61 mg GAE/100 ml; Anthocyanins (ANC): 44 mg/100 ml	(37)
Cashew nut	~5 g/100 ml	Acetic 2.9 g/L; Latic 0.17 g/L; Glucoronic 2.9 g/L; Citric 0.70 g/L	–	–	(38)
Coffee	7.2 g/L	–	–	TPC: 543 mg GAE/L; 5–caffeoylquinic acid (5-CQA) 175 μL/ml	(35)
Acerola (AC), guava (G) and tamarind (T) by-products	AC = 0.8%; G: ~0.68%; T ~0.84% (acetic acid m/v)	Acetic (g/L): AC 14.6, G 7.3, T 5.0; Glucoronic (g/L) AC: 14.6, G and T: 11.7; Latic (g/L) AC: 3.16; Citric (g/L): G 2.6; Ascorbic (g/L) AC: 3.28, G 0.14	–	–	(31)
Bacuri (B), Taperebá (T), and Cupuaçu (C)	B: 0.41 g/L; T: 0.39 g/L; C: 0.49 g/L	–	B: 2.27 g/L; T: 3.51 g/L; C: 5.07 g/L	TPC (mg GAE/L): B 38.3, T 34.9, C: 33.4	(40)
Green tea and Yerbá-mate with strawberry guava	3.20–3.28 g/L	–	–	TPC: ~80–184 mg GAE/L	(33)
Yerba-maté with sucrose (S), Borá (B), Mandaguari (M), and Apis (A) honey	B: 11.2 g/L; S: 10.5 g/L; M: 10.3 g/L; A: 8.8 g/L	–	–	TPC (mg GAE/L): B 59.2, S 52.5, M 48.0, A 51.2	(34)
Passion fruit (P) and Apple (A)	P: 1.05%, A: 0.65% (acetic acid m/v);	Ascorbic: P and A 0.78 mg/100 g	P: 6.2%; A: 1.65%	TPC (mg GAE/100 g): P 13.2, A 29.3; ANC (mg/100 ml): P 0.07, A 0.06; Flavonoid (mg/100 ml): P 0.78, A 1.33	(21)
Passion fruit (*Passiflora edulis*) tea	–	Acetic: 3.1 g/L	0.72 g/L	TPC: ~160 mg GAE/L; Flavonoid ~80 mg QE/L	(24)
Cocoa beans (CB)	~0.1–0.3 mg/100 ml	–	0.00%	~0.8–2.3 mg GAE/ml	(29)
Yerba-maté (Y) and Yerba-maté with pitanga pulp (YP)	–	Acetic: ~8 g/L; Glucuronic: ~1.2 g/L; Ascorbic: Y ~0.3 g/L, YM ~0.9 g/L	–	TPC: 840–860 mg GAE/L Flavonoids (mg CAT/L): Y ~190, YM ~410	(28)
Red-araçá (R), Butiá-da-serra (B), and Jaboticaba (J)	R ~0.8%, B ~0.5%, J ~1.0% (acetic acid m/v)	Acetic: R and J ~6 g/L; B ~2.5 g/L	R ~5.2 g/L; B ~1.0 g/L, J ~1.8 g/L	TPC (mg GAE/100 ml): R ~25.2, B ~17.5, J ~20.0	(43)

In the case of using fruits in the fermentation process for kombucha analogs production, red-araçá, butiá-da-serra, and jaboticaba (43) presented final pH values between 2.5 and 2.9 after 10 days of fermentation, differing significantly (*p* < 0.05) from day zero to day 5 and remaining constant until the end of fermentation (10 days). Passion fruit and apple (21) showed final values between 3.15 and 3.50 after 10 days of fermentation, with a significant difference (*p* < 0.05). Finally, pitanga and umbu-cajá (17) showed final values of 2.67 and 2.09, respectively, after 7 days of fermentation, differing significantly (*p* < 0.05) from the initial pH values. In the case of umbu-cajá, the value was lower than that established by legislation; however, at the beginning of fermentation, the pH was already lower than that permitted by legislation (2.19). Therefore, a correction may be necessary to ensure the final beverage is not overly acidic. In Junior's study (17), the authors also found that pitanga had a higher quantity of citric acid, while the others presented a higher quantity of acetic acid. When using fruit by-products, Leonarski et al. (30) reported final pH values between 2.49 and 2.58 after 15 days of fermentation using acerola (differing significantly (*p* < 0.05) from the initial values). In contrast, for acerola, guava, and tamarind by-products (31), final values between 2.78 and 3.21 were reported after 7 days of fermentation. When using grape and mango peels (23) in the second fermentation (48 h), the authors reported final pH values between 3.3 and 3.5. In Leonarski (30) study, which verified sugar consumption, samples made with 5%, 3%, and 1% (w/v) acerola by-product reported consumption rates of 56.6%, 43.4%, and 31.6%, respectively.

When using coffee as an extract for fermentation, Arabica coffee (35) was used after 21 days of fermentation, and the final pH value was 3.25. A significant decrease (*p* < 0.05) was observed on the 6th day, after which it remained constant until the end of fermentation. For Cascara coffee (20), it varied from 3.7 to 3.5 at the end of 9 days of fermentation (no statistical difference, *p* > 0.05); while for coffee leaf and coffee leaf-toasted mate (36), the values varied from approximately 4.0 to 3.4–3.9 at the end of 9 days of fermentation, with a significant difference (*p* < 0.05) only for kombucha with coffee leaf (final pH 3.4). The pH decreases with fermentation time but tends to reach equilibrium at some point, not decreasing significantly over the course of the days. Regarding sugar consumption, it was observed that for the 21-day fermentation, there was a 24% reduction in sugars, while for the studies with 9 days of fermentation, consumption varied between 20 and 37%.

In general, regardless of the raw material, it was possible to ferment kombucha-type beverages. In the case of yerba mate, it is necessary to monitor pH values to ensure that they remain within the limits established by legislation at the end of fermentation. Additionally, the inclusion of other raw materials can be an alternative for pH control, as reported in previous studies. When using fruits or fruit by-products, it is necessary to assess the acidity of the raw material to control the pH during fermentation and prevent the product from becoming too acidic. When using coffee, it appears that even in longer fermentations, it is possible to maintain the pH value within the limits established by legislation.

## 5 Kombucha bioactive compounds

### 5.1 Phenolic compounds

As previously reported, flavoring agents such as fruit pulps, herbs, or spices can be added after the second fermentation to improve both the sensory profile and the bioactive composition of kombucha. When these ingredients are combined with the tea base, their own phenolic compounds mix and react with tea polyphenols, which may influence the release, transformation, or increased availability of bioactive molecules during fermentation. The amount of phenolic compounds in the beverage during fermentation is influenced by several factors, including the type of tea and flavoring agents used, sugar concentration, fermentation time, temperature, and the concentration of flavoring agents (11). Moreover, several studies have reported increases in phenolic content during fermentation, although the mechanisms responsible for these changes are not yet fully understood and may vary according to fermentation conditions and substrate composition. For instance, Leonarski et al. (18) observed a 13.1% increase in total phenolic content (TPC) (determined by Folin-Ciocalteu method) during the first 3 days of fermentation, which then remained stable (no significant difference, *p* > 0.05) until day 15 in green tea kombucha (without flavoring agents). Similarly, Lopes (32) reported an increase in TPC values, quantified using Folin-Ciocalteu method, from 774.8 to 831.7 mg GAE (Gallic Acid Equivalent)/L, after 12 days of fermentation in yerba-maté kombucha. This behavior was also observed in the study by Suna et al. (46) for green tea kombucha fermented for 5 days. In the study by Jakubczyk et al. (47), the authors observed a gradual increase (with a significant difference, p < 0.05) in the total phenolic compound content of green tea kombucha throughout fermentation (14 days).

In a study conducted by Noronha et al. (19), the phenolic profile of black tea kombucha was identified and quantified (based on relative quantification) over 10 days of fermentation using UPLC-MS. The beverage exhibited a rich composition of flavonoids, including (–)-epicatechin, (+)-catechin, quercetin 3-O-glucoside, quercetin, and kaempferol, which were among the most abundant compounds. Phenolic acids such as gallic, vanillic, gentisic, syringic, p-coumaric, ferulic, and 4-hydroxybenzoic acids were also detected. Overall, no clear trend of increasing or decreasing abundance was observed for the specific phenolic compounds; instead, their behavior varied throughout fermentation, with some compounds increasing in abundance while others decreased. For example, the abundance of flavonoids generally remained stable, showing little variation over time. For (–)-epicatechin minimal change in the relative quantification from day 0 to day 10 was observed. A similar pattern was observed for catechin 5-O-gallate, whose abundance remained essentially constant between day 0 and day 10. Conversely, several phenolic acids exhibited a clear tendency to increase in abundance during the fermentation process. Gallic acid, the phenolic acid present at the highest levels, increased of 36% on day 10. Syringic acid followed a similar trend, with an increase of 132% over the same period, both estimated based on relative quantification.

A possible explanation for those phenomena is the enzymatic activity of the microbial consortium under acidic conditions, which may contribute to the hydrolysis of complex polyphenols into smaller, more bioavailable compounds. However, this does not significantly impact flavonoids. Their relative stability throughout fermentation may be attributed to their chemical structure, which is generally more resistant to microbial or acid-induced degradation, resulting in minimal changes in abundance over time (30, 32).

Exotic fruits such as pitanga (*Eugenia uniflora* L.) and umbu-cajá (*Spondia tuberosa*) are abundant in Northeast Brazil and have attracted increasing attention from the scientific community due to their high levels of bioactive compounds, antioxidant capacity, and unique sensory properties. Júnior et al. (17) evaluated the phenolic composition of kombucha formulations prepared with pitanga and umbu-cajá and compared them with traditional kombucha. The authors reported a high concentration of epigallocatechin gallate (EGCG), corresponding to 63.69%−76.84%, and of caftaric acid (7.19%−11.72%) (values obtained based on relative percentage from the total value of the phenolic compounds). The predominance of EGCG, in both studies: Noronha et al. (19) and Júnior et al. (17) is attributed to the use of green tea leaves in the preparation of kombucha, as they are a major source of this bioactive compound. In agreement with the findings of Noronha et al. (19), no significant change in EGCG concentration was observed between day 0 and day 7 in traditional kombucha. In contrast, formulations enriched with pitanga and umbu-cajá showed a slight decrease in EGCG levels during fermentation, from 76.8 to 68.4% and from 72.1 to 68.04%, respectively.

Phenolic concentrations can fluctuate during fermentation, with cycles of rise, decline, and re-increase influenced by the substrate, microbial activity, and fermentation stage. For example, this fluctuating pattern was observed by Santos et al. (34) in kombucha produced with Yerba maté (a traditional beverage originating from the Southern Part of Brazil, specifically Rio Grande do Sul) and *Apis* honey. The authors reported a slight increase in all quantified compounds on day 5, followed by a decrease on day 10 and a subsequent rise on day 15, which was then sustained until the end of fermentation. Despite these oscillations, after 20 days the Yerba maté/*Apis* honey kombucha showed a significant increase in several key compounds, including 3-CQA (3-caffeoylquinic acid), 5-CQA (5-caffeoylquinic acid), 4-CQA (4-caffeoylquinic acid), 3,5-DQA (3, 5-dicaffeoylquinic acid), 4, 5-DQA (4, 5-dicaffeoylquinic acid), rutin, caffeine, and 3,4-DQA (3,4-dicaffeoylquinic acid).

In the development of unconventional kombucha beverages, particularly those produced from phenolic-rich Brazilian raw materials, it is essential to assess the bioaccessibility of phenolic compounds, as their initial concentration in the substrate does not necessarily reflect the fraction that remains stable and available for absorption after gastrointestinal digestion (48). From a market standpoint, this assessment is equally critical because functional claims, especially those related to bioactive compounds, must be supported by evidence that these compounds remain bioaccessible and physiologically relevant, ensuring credibility, regulatory compliance, and consumer trust (49).

During digestion, phenolics may bind to matrix components such as proteins, minerals, and polysaccharides, or undergo enzymatic and pH-driven degradation, altering their stability and release (48). This phenomenon was demonstrated by Júnior et al. (17), who showed that some compounds are markedly more sensitive to digestion than others. The authors used the standardized static *in vitro* digestion method suitable for food, as described by Minekus et al. (50). Although the beverages exhibited high epigallocatechin gallate content, this compound was absent from the bioaccessible fraction after *in vitro* digestion of umbu-cajá and pitanga kombuchas (ranging from 68 to < 5%). In contrast, other phenolics, such as caftaric acid (from 10.57–10.65 to 22.38–29.98%), catechin (from 2.45–2.23 to 17.61–23.48%), and hesperidin (from 1.48–1.63 to 22.43–28.47%), were consistently the most bioaccessible compounds, showing levels even higher than non-digested samples. According to the authors, these results stem from compound-specific structural stability under simulated gastrointestinal conditions, including the partial hydrolysis of polymerized flavonoids and chemical rearrangements that enhance the release of certain phenolic acids.

Since various types of raw materials were used, it is interesting to determine the total content of phenolic compound classes that may be present in the beverages. Some studies analyzed flavonoids, anthocyanins (subgroup of flavonoids), and tannins (Tables 3, 4). For traditional kombuchas (green or black tea), flavonoids (21, 24, 25) and a low concentration of anthocyanins (0.06 mg/100 mL) were identified (21). For kombucha made with blueberry (37) and passion fruit (21), the presence of anthocyanins was confirmed, with the blueberry drink showing higher levels. As for flavonoids, they were identified in beverages containing passion fruit (21, 24), apple (21), and yerba mate (28).

Overall, considering the dynamic behavior of phenolic compounds during fermentation and *in vitro* digestion, further studies under *in vivo* conditions are still necessary to better understand how these transformations influence bioaccessibility, absorption, and potential biological effects.

### 5.2 Antioxidant activity

Numerous studies indicate that Brazilian ingredients commonly incorporated into kombucha formulations, including blueberry (37), acerola (31), cupuaçu, tapereba and bacuri (40) and others regional plant matrices or agro-industrial by-products, exhibit rich phytochemical profiles characterized by high levels of phenolic compounds, flavonoids, anthocyanins, and other bioactive secondary metabolites (24, 37, 40). These compounds are well documented for their capacity to quench reactive oxygen species via mechanisms such as radical scavenging, hydrogen atom donation, and metal-ion chelation, which may contribute to the antioxidant potential observed in these formulations (51).

Studies evaluating the antioxidant potential of kombucha report considerable variability in how this parameter is assessed and presented. While some investigations characterize antioxidant activity kinetically throughout the fermentation process, capturing temporal fluctuations associated with microbial metabolism and polyphenol biotransformation (24, 40), others quantify antioxidant capacity only at discrete time points, typically comparing the unfermented substrate with the fully fermented beverage (18). This methodological heterogeneity contributes to differences in reported trends and underscores the importance of interpreting antioxidant results considering the sampling strategy and analytical design employed in each study.

For instance, Crispino et al. (40) produced kombucha beverages using cupuaçu (295.02 μmol Trolox/ml), taperebá (295.40 μmol Trolox/ml), and bacuri (307.90 μmol Trolox/ml), three Amazonian fruits from Brazil that exhibited promising antioxidant capacity by the DPPH assay. These results were consistent with the high phenolic content characteristic of these fruits. Another study by Lima et al. (24) produced kombucha using *Passiflora edulis* infusion, which exhibited remarkable radical-scavenging activity (RSA), with ABTS values of approximately 80% and DPPH values near 82%, highlighting the potential of *Passiflora edulis*, an abundant resource in tropical and subtropical regions such as Brazil, as a functional ingredient in kombucha formulations.

Lopes et al. (32) reported that the antioxidant activity of kombuchas produced from green tea and yerba-maté increased markedly during 12 days of fermentation (with a significant difference, *p* < 0.05) when measured by the ABTS assay (from 16.2 to 31.3%). In contrast, the increase observed with the DPPH method was modest (only 3.7%). This difference may be attributed to the ABTS assay's greater sensitivity to a broader range of phenolic compounds (polar and non-polar) released or formed during fermentation. In contrast, DPPH reacts with a more limited set of polar antioxidants (51). Thus, the stronger increase observed with ABTS likely may reflects enhanced release and biotransformation of more polar phenolic compounds throughout fermentation, which was corroborated by the authors with TPC results. Another study by Leonarski et al. (30) reported a 14.8% increase (with a significant difference, p < 0.05) in the antioxidant capacity, measured by the DPPH method, in kombucha formulated with 3% acerola by-product after 15 days of fermentation.

Moreover, the antioxidant activities reported in kombucha studies are commonly corroborated by the beverage's phenolic profile, which is typically characterized by high-performance liquid chromatography (HPLC). This analytical and robust approach enables the identification and quantification of individual phenolic compounds and other bioactive metabolites, providing a more reliable support for the trends observed in spectrophotometric assays and helping to explain variations in antioxidant capacity throughout fermentation. For example, a study by Júnior et al. (17) produced kombucha with outstanding antioxidant capacity using pitanga (2,350.6 and 474.5 μmol Trolox/100 mL for DPPH and ORAC, respectively) and umbu-cajá (2,361.91 and 296.6 μmol Trolox/100 mL for DPPH and ORAC, respectively), both abundant in Northeast Brazil. The antioxidant activities were discussed by the authors in association with the diverse phenolic compounds identified and quantified by HPLC, including syringic acid, hesperidin, procyanidin B1, catechin, procyanidin B2, caftaric acid, rutin, among others.

Other important point to consider is how these antioxidant activities are modulated by gastrointestinal digestibility, since the bioaccessibility of phenolic compounds determines whether their antioxidant effects persist throughout the digestive process and reach the intestinal compartments where absorption occurs. Studies using simulated digestion models indicate that the stability, release, and transformation of phenolics can substantially influence the post-digestion antioxidant capacity, providing a more realistic perspective on the potential physiological relevance of bioactive compounds from Brazilian kombucha. This evidence can be observed in the study by Júnior et al. (17), who evaluated the bioaccessibility of antioxidant capacity by *in vitro* digestion after simulated digestion and reported a substantial decrease in activity by DPPH method (pitanga = 2,350.6 to 12.99 and umbu-cajá = 475.5 to 10.05 μmol Trolox/100 ml). According to the authors, this behavior may be influenced by the high reactivity of phenolic compounds, which are susceptible to degradation by oxygen and ions under gastrointestinal conditions. In addition, these compounds may interact with other matrix components or undergo enzymatic degradation during digestion, further reducing their detectable antioxidant activity.

Considering the above, these investigations suggest that fruits and Brazilian agro-industrial by-products can serve as important sources of antioxidant compounds in kombucha formulations. Nevertheless, antioxidant activity in Brazilian kombucha studies is predominantly assessed using DPPH, RSA, ABTS, and ORAC assays. Approaches addressing other antioxidant mechanisms (e.g., metal chelation, reducing power, inhibition of lipid peroxidation, and cellular antioxidant activity) and *in vivo* evaluations remain scarce in the literature, representing important research gaps, together with the need for studies to substantiate these effects and to explore other relevant biological activities.

## 6 Sensory analysis of traditional and non-traditional kombucha

While characterizing the phytochemical composition and bioactive properties of kombucha formulated with Brazilian flavors provides important insights into its functional potential, sensory evaluation is equally essential for developing new formulations incorporating regional ingredients during the kombucha fermentation. Sensory analysis enables researchers and producers to determine how these additives modulate key sensory attributes, such as aroma, flavors, and overall liking, and to assess consumer acceptability, thereby guiding formulation optimization and supporting the creation of products that align with market expectations.

The sensory evaluation of Brazilian kombucha was assessed by a wide range of studies. For instance, Câmara et al. (31) produced kombucha incorporating flavors obtained from Brazilian agro-industrial by-products (acerola, tamarind, and guava) to valorize these residues and expand the sensory diversity of the beverage. Through descriptive sensory analysis (Multiple comparisons of check-all-that-apply, CATA) combined with purchase-intention testing, with a group of 60 untrained panelists, 36 women and 24 men aged between 18 and 65, the authors observed that the acerola formulation was predominantly associated with citrus-like notes, the tamarind formulation with stronger vinegar-like attributes, and the guava formulation with a sweeter sensory profile. The sour and citrus tastes were the attributes with the highest perceived intensity (87%), followed by the sweet aroma attributes (79%) and the vinegar taste (69%). The purchase-intention scores averaged 3.7 for guava kombucha, 3.3 for tamarind, and 3.2 for acerola, indicating a higher consumer preference for the guava-based product, which differed significantly from the other samples at the 5% significance level. Collectively, the results demonstrated that the guava formulation performed superiorly across the evaluated sensory parameters, a finding potentially linked to established consumer familiarity and preference for guava flavor.

Another approach was studied by Lima et al. (24), which investigated the sensory properties of kombucha produced using a Brazilian *Passiflora edulis* leaf infusion as an alternative substrate to conventional green tea. A panel of 60 untrained consumers assigned comparable scores to both beverages across most sensory attributes; however, the *Passiflora edulis* formulation demonstrated a slight advantage in flavor (4.21 vs. 3.84 for green tea) and overall acceptance (3.88 vs. 3.86), with no significant differences observed (Student's *t-test, p* > 0.05), along with a higher purchase intention (29% vs. 22%).

Considering that the sensory attributes of kombucha change dynamically throughout fermentation, Sales et al. (20) investigated how the use of Brazilian coffee cascara tea influences this progression by assessing odor, taste, flavor, mouthfeel, and appearance on days 3, 6, and 9 of fermentation. One hundred and fourteen panelists (61% female and 39% male) participated in the Acceptance, Purchasing Intention, and Rate All That Apply (RATA) tests. The early stage (day 3) was characterized by a relatively simple sensory profile, dominated by berry-like, woody, and herbal odor notes and by sweetness in taste, reflecting limited acidification and the initial extraction of volatile compounds from the cascara. As fermentation advanced, particularly by day 6, the beverage developed a markedly more complex profile, with the emergence of flowery, yellow-fruit, and raisin/prune aromas and a shift toward sourness and bitterness in taste. These changes were accompanied by the appearance of acetic/vinegar, ripe-fruit, and syrup-like flavor notes, as well as astringency in mouthfeel, attributes consistent with increased acid production and microbial metabolism during the mid-fermentation phase. Despite these transformations, consumer acceptance and purchase-intention scores remained relatively stable (with no difference significative, p > 0.05), with values of 6 and 3 on days 3 and 9, respectively, suggesting that the sensory evolution throughout fermentation did not strongly influence consumer willingness to consume or purchase the product.

Another study conducted by the same authors (36) investigated the acceptance of kombucha produced with a 50:50 blend of coffee leaf tea and toasted maté tea. Sensory evaluation, combined with cluster analysis, enabled the authors to associate acceptance patterns with consumer behavior. 103 panelists participated in the Acceptance, Purchasing Intent, and Rate All That Apply (RATA) analysis. For *Coffea arabica* leaf tea kombucha (CL), the most intense aroma and flavor descriptors included fruity, peachy, sweet, and herbal notes, while kombucha made from *Coffea arabica* leaf tea with toasted maté (CL-TM) infusion featured additional notes of roasted mate. The highest acceptance scores were given to CL-TM and CL on day 3 (6.6 and 6.4, respectively, on a nine-point scale), with no significant difference (*p* > 0.05). This consumption pattern suggests a greater familiarity with beverages that exhibit stronger acidity, effervescence, and complex flavor profiles, which likely contributed to their higher preference for this kombucha.

In summary, considering that sensory acceptance is strongly influenced by the fermentation stage and consumers' prior beverage habits, sensory analysis is essential for developing new kombucha flavors. It enables the identification of how different ingredients and fermentation conditions shape key attributes, such as aroma, taste, after-notes, and mouthfeel, ensuring that formulations align with consumer expectations. This integration between sensory perception and organoleptic characteristics results in kombuchas that are more appealing and better suited to the sensory profiles valued within the Brazilian context.

## 7 Challenges of kombucha production in Brazil

Brazil was a pioneering country in introducing legislation (6) for kombucha production, as briefly reported in the introduction section. In addition to the parameters of pH, volatile acidity, alcohol content and pressure (atm at 20 °C) in kombucha added with CO_2_, the legislation also addresses the definition of kombucha, mandatory and optional ingredients, label classification (alcohol content, pasteurization), prohibition of mentioning possible functional and health claims (since more clinical studies in humans are still needed) among other information. While Brazilian legislation specifies a maximum pH of 4.2 for the final product, in the United States, according to the FDA Model Food Code, this critical value (4.2) must be reached within 7 days of fermentation. A failure to achieve the pH may indicate contamination, and the product should be discarded (52). For Canada, the same requirement was also presented by the BC Center for Disease Control (BC-CDC) in the “Food Safety Assessment of Kombucha Tea Recipe and Food Safety Plan” (53).

According to Martin et al. (10), although legislation has been important in regulating and promoting kombucha production in the country, some challenges are faced in implementing these rules. Among them, we can highlight: (a) the mandatory use of *C. sinensis* in the fermentation process, that is, if other teas (such as yerba mate) are used in the first fermentation, the product cannot be called kombucha. (b) The mandatory use of SCOBY (a term used to refer to the cellulosic film that housed the community of bacteria and yeasts) is also necessary according to legislation, although it has been verified that using only a portion of previously fermented beverage (popularly known as “starter tea”) is sufficient to carry out the fermentation of the beverage. However, the legislation itself does not define the cellulosic part as SCOBY; therefore, the use of the liquid inoculum is sufficient to classify the product as kombucha. (c) Raising public awareness of the real benefits of the beverage, demystifying its possible probiotic power until this is properly proven. (d) Acceptance of the pasteurization process as an important thermal process for sterilizing the product (if flavorings are added, for example) and inactivating microorganisms and controlling CO_2_ production, to extend the shelf life of the beverage and reduce commercial losses and possible accidents. (e) Control of ethanol production and review of volatile acidity limits, since production varies according to microorganisms and process parameters.

Regarding the production of ethanol in beverages, Rossini et al. (54) conducted a study on the alcohol content of beverages marketed in Brazil. Before the standard (6) was implemented, 97% of the beverages evaluated did not comply with the legislation, as they had an alcohol content higher than 0.5% (v/v). After the implementation of the legislation, non-compliance was still observed in 67% of the beverages evaluated. These results highlight the importance of monitoring and standardizing the beverage production process to meet the parameters established by legislation. In the United States, non-alcoholic beverages are subject to regulations by the Food and Drug Administration (FDA). According to the legislation, non-alcoholic beverages must not contain more than 0.5% alcohol by volume (ABV) at any time during production, bottling, or after bottling (55). For Canada, alcohol labeling is required when the product exceed 1.1% alcohol by volume (ABV), and for BC-CDC, 1.0% ABV (53).

Another important factor to consider in implementing legislation is information about the flavoring process or even secondary fermentation. As reported in some studies (17, 23, 38, 40), the addition of flavoring (like pulp, nectar, juice, and others) is performed as a secondary fermentation step, which has a significantly shorter duration (up to approximately 72 hours) compared to the initial fermentation. Aiming for greater standardization in product development, addressing this stage of the process would be important for better controlling beverage production.

## 8 Conclusion

Brazil presents itself as a promising country to produce kombucha-like beverages using alternative extracts, mainly due to its extensive biodiversity and wide availability of native and tropical fruits. Among the alternative substrates investigated, fruits were the most used, generally exhibiting fermentative behavior similar to that of traditional kombucha, including pH reduction and the production of organic acids and ethanol. Several studies have also observed increases in total phenolic content and antioxidant activity after fermentation. However, the mechanisms responsible for these changes are not yet fully understood, since the bioactivity of kombucha-type beverages can be influenced by microbial metabolites, organic acids, and biotransformation products generated during fermentation.

Future studies should focus on better understanding the biochemical and microbiological mechanisms involved in the fermentation of alternative substrates, particularly the transformation of phenolic compounds and their relationship with antioxidant activity. In addition, the standardization of alternative raw materials remains a challenge, as seasonal and regional variations may affect fermentation kinetics, beverage composition, sensory characteristics, and storage stability. Therefore, further studies involving metabolomic analyses, microbial community characterization, shelf-life evaluation, sensory acceptance, and industrial-scale production are essential to support the commercialization of kombucha-type beverages produced from alternative extracts.

Regarding regulatory aspects, Brazilian legislation already includes important regulations for kombucha production; however, clearer and more comprehensive guidelines are still needed for kombucha-type beverages produced from non-traditional substrates. Future regulatory updates should include more specific standards regarding identity, composition, labeling, microbiological safety, alcohol content, and quality control parameters throughout the entire production chain.
